# Transcriptional regulation at a glance

**DOI:** 10.1186/1471-2105-8-S6-S2

**Published:** 2007-09-27

**Authors:** Silke Sperling

**Affiliations:** 1 Max Planck Institute for Molecular Genetics, Ihnestrasse 73, 14195 Berlin, Germany

## Abstract

Considering that 80 genomes have been sequenced, providing us with the static information of the genome, it is still a long way to reveal the relationship between complex genotypes and phenotypes. The transcriptional regulation process is one of the obstacles that need to be understood to bridge our current information gap. It describes the first step from the genomic sequence information to RNA templates used for protein production or as direct functional units, like non-coding RNAs (e.g. micro RNAs). This introduction aims to highlight the key aspects of the transcriptional process from our current understanding.

## Introduction

**Figure 1:**
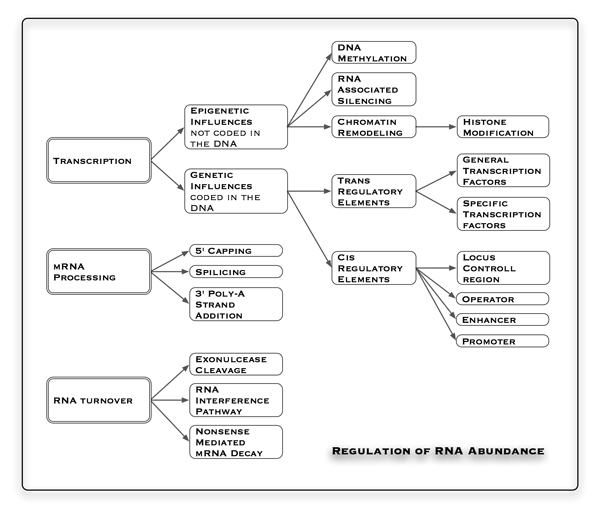
**RNA abundance in a cell**. Shown is an overview of factors regulating RNA abundance in a cell.

The transcriptome as the pool of all transcribed elements in a given cell varies depending upon its context. Looking at gene expression in mammalian organisms several spatial and temporal restrictions occur. RNA can be transported as a ribonucleoprotein (RNP) particle to specific cellular locations, e.g. tau mRNA in neurons, leading to a location gradient of RNA within a single cell. Many genes show transient expression at particular developmental stages (e.g. globin genes), at specific times in the cell cycle (e.g. histone genes during S phase) or in response to environmental signals. This implies that the expression of a gene is highly coordinated and influenced by a broad panel of external and internal cellular factors (Figure 1). This review aims to highlight the key aspects and for advanced reading see additional file 1.

## RNA factory

In eukaryotes RNA synthesis is catalyzed by three different RNA polymerases depending on the type of RNA produced. RNA polymerase II promotes the synthesis of the pre-mRNA coding for proteins. It consists of twelve subunits, where the largest subunit contains an essential carboxyl terminal domain that is phosphorylated during initiation. The majority of RNA polymerase II promoters are characterized by a TATA box approximately 25 to 35 base pairs upstream of the transcriptional start site (TSS). Frequently genes transcribed at a low rate coding for housekeeping genes are marked by a GC-rich stretch of 20–50 nucleotides within the first 100 bp upstream of the TSS and are in part recognized by the transcription factor SP1. The transcriptional initiation by RNA polymerase II at almost all promoters requires a set of several general multi-protein transcription factor complexes (TFIID, TFIIA; TFIIF; TFIIE; TFIIH). TFIID contains the TATA box-binding protein, and TFIIH the helicase to separate DNA strands and kinase to phosphorylate the carboxylterminal domain of the RNA polymerase II.

Although transcription is the first and probably most highly regulated step in expression, it is usually only the beginning of a series of events required to produce a functional RNA. Primary transcripts of eukaryotic mRNAs undergo extensive modifications, such as 5'capping, splicing and 3'poly-A strand addition. These RNA processing steps are tightly coupled to the transcriptional elongation and the promoting factors are mainly bound to the phosphorylated tail of the RNA polymerase II. As soon as RNA polymerase II has produced about 25 nucleotides of RNA, the 5'end of the new RNA molecule is modified by addition of a "cap" that consists of a modified guanine nucleotide. This landmark helps the cell to distinguish mRNAs from other types of RNA molecules present within the cells and is bound by the cap-binding protein complex (CBC). The CBC promotes proper processing and finally the export of RNA.

RNA splicing is the critically important step in which the different portions of a eukaryotic protein coding sequence are joined together by removing transcribed intron sequences. In human transcripts around 60% of genes are spliced in a variety of ways to produce a set of different mRNAs, thereby allowing a corresponding set of different proteins to be produced from the same gene, which can also be cell type specific, e.g. α-tropomyosin in striated muscle, smooth muscle, fibroblast and brain. Splicing is performed largely by RNA molecules instead of proteins such that specific RNA molecules (small nuclear RNAs, snRNAs) recognize intron-exon borders and participate in the chemistry of splicing. Each snRNA is complexed with at least seven protein subunits to form a snRNP (small nuclear ribonucleoprotein) that forms the core of the spliceosome. The assembly of the spliceosome occurs as pre-mRNA is emerging from a transcribing RNA polymerase II. Several components of the spliceosome are carried on the phosphorylated tails of RNA polymerase. Their transfer directly from the polymerase to the nascent pre-mRNA helps the cell to keep track of introns and exons. Furthermore, the exon sizes are much more uniform than intron sizes, averaging about 150 nucleotide pairs across a wide variety of eukaryotic organism. A group of spliceosome components, called SR proteins, assemble on exon sequences and mark each 3' and 5' splice sites. If splice-site selection were determined solely by the snRNPs acting on a preformed, protein-free RNA molecule, mistakes such as exon skipping and the use of cryptic splice sites would be very common.

Consensus nucleotide sequences of the RNA at the 3'end encode the cleavage and polyadenylation signal for the cleavage stimulation factor (CstF) and cleavage/polyadenylation specificity factor (CPSF), which travel to the 3'end processing sequence at the RNA polymerase II tail. After the 3'end is cleaved, the RNA polymerase II continues to transcribe, in some cases as many as several hundred nucleotides beyond the DNA that contains the 3' cleavage-site information. However, the polymerase soon releases its grip on the template and transcription terminates. The piece of RNA downstream of the cleavage site is then degraded in the cell nucleus. Potential triggers that stop polymerase II processivity after RNA cleavage are a conformation change of the polymerase due to the transfer of the 3'end processing factors from the polymerase tail or feedback signaling due to the lack of the cap structure on the 5'end of the RNA that emerges from the polymerase.

## RNA turnover

In eukaryotes the transcriptional process is physically separated from the translational process (nucleus, cytoplasm) and the mRNA transport into the cytosol as well as the mRNA stability are highly controlled. Usually an mRNA must be marked by the appropriate set of proteins to be transported, like the cap-binding complex being bound and others like specific snRNP proteins being absent. Other proteins, placed on the RNA during splicing, mark exon-exon boundaries and signify complete splicing events. In eukaryotic cells, mRNAs are quite stable and some, like the β-globin mRNA, have half-lives of more than 10 hours, however, many have half-lives of 30 minutes or less. Unstable mRNAs often code for regulatory proteins, such as growth factors and gene regulatory proteins, whose production rates have to be changed rapidly. In contrast, the vast majority of mRNAs in a bacterial cell are very unstable, having a half-life of about 3 minutes, and this high turnover enables the quick adaptation to environmental changes. The nuclear decay of RNA involves both 3' to 5'and 5' to 3' exonucleases, where the first is the best studied mechanism and forms a complex termed the exosome. A significant RNA turnover occurs co-transcriptionally, probably at a number of stages during initiation and elongation by RNA polymerase II. Another aspect of RNA surveillance plays the handling of double-stranded RNAs, produced by inadvertent bidirectional transcription. Such RNAs are subjected to extensive adenosine-to-inosine editing by enzymes called ADARs (adenosine deaminase acting on RNA), which destroy their double-helical character and ability to enter the RNA interference pathway. Short interfering RNAs (siRNAs) and microRNAs (miRNAs) are components of the RNA based mechanism of gene regulation at the transcriptional and post-transcriptional level respectively, which leads to cleavage of suspected target RNAs. Once in the cytoplasm, mRNAs are further subjected to translation-dependent surveillance. Here a process called nonsense-mediated mRNA decay (NMD) provides a mean to degrade abnormal mRNAs that encode potentially deleterious truncated proteins. Additionally, an estimated one-third of naturally occurring, alternatively spliced mRNAs are also targeted for NMD, potentially providing an additional mechanism to achieve proper levels of gene expression.

## Chromatin remodeling

DNA is packaged into chromatin thereby constraining the size of the molecule that is approximately 2 meters of DNA per human cell. Chromatin represents a repeating unit of histones and DNA that form the nucleosome. Highly condensed chromatin (heterochromatin) is transcriptionally silent. Simplistically, there are three major levels of chromatin organization: nucleosomes as "beads on a string", 30 nm chromatin fiber consisting of packed nucleosome arrays and highly condensed metaphase chromosomes. In general, 146 bp of DNA are wrapped around a nucleosome core consisting of an assembled histone H3-H4 tetramer and two H2A-H2B dimers. The interaction of DNA with the positively charged histones is made through the negatively charged DNA backbone. Histones are highly conserved, and H3 and H4 are even the most highly conserved proteins in nature. Approximately 60 million molecules of each histone are present in a human cell. The majority of histones in proliferating cells are synthesized during the S phase of the cell cycle, when they are rapidly deposited onto newly replicated DNA. Disruption of this replication-dependent nucleosome assembly in humans triggers spontaneous DNA damage, S phase arrest and chromosome rearrangements, where the latter is a prominent feature of many human cancers. However, excess histone accumulation is harmful to cells because they bind non-specifically to DNA, thus interfering with many processes. Mechanisms of how histone degradation and abundance are controlled are currently a field of major interest.

To gain access to the DNA for transcription factors and the transcriptional machinery with the RNA polymerases, the DNA needs to be unwrapped from the histone core. Several methods of chromatin remodeling have been proposed, such as deposition of histones by chaperones, assembly and reassembly of nucleosomes by ATP-dependent factors and DNA translocation enzymes. Further specific patterns of modification of histone tails attract or repel regulatory proteins of the chromatin remodeling complex. Histone modifications can influence one another and thus, not just the level of modification but also the pattern may dictate biological outcome. The main modifications are acetylation and methylation, followed by phosphorylation. A panel of histone acetyl transferases (HAT), histone deacetylases (HDAC) and histone methylases (HMT) has been identified that intricate gene regulation. Histone acetylation is so far the best studied modification, here an acetyl group is covalently bound to basic amino acids in histones and neutralizes the positive charge of histones. Therefore, ionic interaction between the histone core and DNA is decreased leading to a decrease in chromatin condensation. Different modification types, like acetylation of histone 4 or histone 3 could be associated with active transcription of the respective gene. Specific patterns of histone modifications attract or repel regulatory proteins, e.g. bromodomain proteins bind to acetylated lysine and chromodomain proteins bind to methylated lysine residues of the histone tail. Bromodomain containing proteins are components of TAF complexes binding to the TATA-box binding protein (TBP). Transcriptional activators can recruit histone acetylases to initiate transcription and repressors can recruit deacetylases to prevent the access of the transcriptional machinery to the DNA by a higher condensation of the DNA template.

Although non-histone proteins and histone modifications are key features of specialized chromatin domains such as heterochromatin, these domains are also characterized by the incorporation of specific histone variants. The majority of histone variants documented to date correspond to two types of histones, H2A and H3. Among H2A variants, H2A.X is one of the best studied ones, given its specific phosphorylation during DNA damage response. A major H3 variant is CENP-A (centromere protein A), an evolutionary conserved replacement variant that is specific for centromeric chromatin and is essential for centromere function, like chromosomal alignment and segregation.

## DNA methylation

Once differential expression patterns have been set up, epigenetic mechanisms can ensure that differential expression patterns are stably inherited when cells divide. DNA methylation is thought to play a major role in this respect permitting the stable transmission from a diploid cell to daughter cells of chromatin states that repress gene expression. DNA methylation is a covalent modification of the DNA and does not alter the base pairing. It occurs in almost all living organisms, from bacteria to plant and fungi, from invertebrates to vertebrates. Its abundance and role differs among these genomes. The pattern of DNA methylation changes dramatically during mammalian development, such that primordial germ cells consist of mainly unmethylated DNA, which becomes methylated during the development to germ cells. Sperm cells show a higher degree of methylation than egg cells and after fertilization a demethylation process during early embryonic development until the blastocyst stage takes place. During pregastrulation the DNA of somatic cell lineages and of trophoblast lineages gain a *de novo *methylation, whereas the primordial germ cells stay unmethylated, e.g. methylation of globin genes in human embryonic blood cells correlates inversely with the activity of the globin genes.

In eukaryotes DNA methylation is mainly restricted to cytosine residues and occurs predominantly at the dinucleotide CpG. Only about 3% of the cytosines in human DNA are methylated and most can be found in CpG dinucleotides, where 80% of cytosines are methylated. CpG islands, short stretches of DNA that are often less then 1 kb long, contain frequently unmethylated CpG dinucleotides in vertebrates. During the course of evolution, more than 3 of 4 CpGs have been lost, leading to an uneven distribution. This marked overall deficiency of CpG sequences and their clustering into CpG islands in vertebrates could potentially be explained by two findings. Deamination of 5-methylcytosine results in thymidine, which forms mismatches with guanine that are inefficiently recognized by the DNA repair system. However, deamination of cytosine results in uracil that is efficiently recognized by the DNA repair system, exercised and replaced. CG sequences placed in regulatory sequences of genes that are transcribed in germ cells are unmethylated and therefore tend to be retained in evolution. Approximately 30,000 CpG islands are found in mammalian genomes, mostly located at promoters of housekeeping genes and some tissue-specific genes. Methylation of CpGs near tumor suppressor genes like p53 or p16, are often related to silencing of these genes in tumors. DNA methyltransferases are responsible for the maintenance and *de novo *methylation of DNA. DMT1 has preferential activity for hemi-methylated DNA and is essential for the inheritance of methylation patterns through DNA replication. DNMT3A and DNMT3B perform the genome-wide wave of demethylation after fertilization followed by the establishment of a new methylation pattern during implantation. Methylation of DNA essentially leads to a repression of transcription by interfering with the consensus sequence of transcription factors (e.g. CTCF of zinc-finger transcription factors) and through the binding of methyl-CpG binding proteins (MBD). The latter provide an enhanced transcriptional control. For example MBD3 is a component of the nucleosome remodeling and histone deacetylation co-repressor complex (NuRD), which can be recruited to the DNA by repressive transcription factors. MeCP2 is the first cloned MBD and a highly abundant protein that recruits transcriptional co-repressors, interferes with chromatin-modifying enzymes and also regulates the expression of certain genes. Its dysfunction causes the RETT syndrome, a neurodevelopmental disorder affecting mainly females as an X-linked gene.

## Specific transcription factors

Transcription factors (TF) can be grouped as general and gene regulatory factors. General transcription factors are a small group of highly abundant proteins that assemble on the promoters of all genes transcribed by RNA polymerase II, whereas thousands of different low abundant proteins are gene regulatory TFs. Approximately 5–10% of human genes encode gene regulatory proteins, where each is usually present in very small amounts in a cell, often less than 0.01% of the total protein content. The gene regulatory proteins allow the individual genes of an organism to be turned on or off specifically. Different selections of gene regulatory proteins are present in different cell types and thereby direct the patterns of gene expression that give each cell type its unique characteristics. Each gene in a eukaryotic cell is regulated differently from nearly every other gene. Given the number of genes in eukaryotes and the complexity of their regulation, it has been difficult to formulate simple rules for gene regulation that apply in every case.

The DNA sequences affecting the level of transcription are commonly named *cis-*elements and the DNA-binding proteins that change the level of transcription are referred to as *trans*-elements.* Cis*-regulatory elements of transcription can be grouped in core-promoters, distal promoters, enhancers, locus control regions (LCR) or operators. Core-promoters are located very closely to the transcription start site and permit the binding of general transcription factors. Enhancers are characteristic for eukaryotic DNA sequences and promote, together with distal promoters, the specific regulation of genes. Locus control regions are super-enhancers that control the expression of a family of genes. In prokaryotes operators and promoters are the main regulatory DNA sequences bound by regulatory proteins and permit the transcription of one or several genes. The majority of *trans*-regulators are either transcriptional activators or repressors; however, some regulatory proteins can function as both depending on the context, like the presence of co-factors or DNA binding sequences. Transcription factors are characterized by conserved structural motifs. Activation domains are thought to stimulate transcription by interacting with general transcription factors in order to assist the formation of the transcription complex on the promoter. DNA-binding domains are necessary to permit specific binding of the TFs to its target genes. A number of conserved structural motifs have been identified that are common to many different TFs with quite different specificities, such as helix-turn-helix proteins, homeodomain proteins, proteins with zinc finger or leucine zipper motifs and helix-loop-helix proteins. Many DNA-binding proteins function as dimers and bind to symmetrical DNA sequences. Even though the amino-acid conservation of particular domains through evolution might be low, they can have a very high degree of structural conservation, like the two homeodomain proteins alpha2 of yeast and engrailed of drosophila that are separated by more than a billion years of evolution.

Transcriptional regulation is based on a modular construction of *cis*-elements and combinatory control by *trans*-elements. Multiple TFs can bind simultaneously to the regulatory sequences and act together on the transcription of a gene. When several transcriptional activators are bound to the regulatory sequences upstream of the promoter, the increase in transcription rate is higher than expected from an additive effect. Thus transcriptional synergy is typically observed between different activators. Transcriptional regulation is generally achieved by tuning the amount or activity of TFs, by interference with the RNA polymerase binding or initiation, or by alteration of the chromatin structure. Enhancers are essential for the rate of transcription that would otherwise frequently only be at low basal levels. Enhancers are not only active when placed just upstream of the promoter, but also when inserted up to several kilobases either upstream or downstream from the transcriptional start site. They can be active in either forward or backward orientation. Gene repressors can function through competitive DNA binding with activators, by masking of the activation DNA surface or direct interaction with general transcription factors.

The existence of gene regulatory proteins became first evident in bacteria with the lambda repressor encoded by bacteriophage lambda. It shuts off viral genes coding for protein components of new virus particles and thereby enables the viral genome to remain a silent passenger. Generally, gene regulatory proteins bind to the major groove of the DNA, where the pattern of attractive force represented by the edges of each base pair show a higher degree of diversity than at the minor groove. The surface of such regulatory proteins is extensively complementary to the special features of the double helix in the recognized sequence. Most proteins make a large number of contacts with DNA where each individual contact is weak but all together ensure a tight interaction. The binding of proteins to DNA is mainly driven by Van der Waals contacts (69,45%) followed by hydrogen bonds (15%) and water mediated bonds (14.5%). Hydrophobic attractions are rarely important (0.05%). Beside base specific interactions, a high number of interactions with the phosphate and sugar residues of the DNA backbone ensure the stabilization of the protein-DNA complex.

## Emerging biotechnologies

Several methods are commonly used to analyze DNA-protein and protein-protein interactions, such as DNA footprinting, electrophoretic mobility shift assays (EMSA), chromatin immunoprecipitation (ChIP), DNA affinity chromatography and two-hybrid systems. Reporter gene assays in cell culture or transgenic animals allow *in-vivo *studies of transcriptional activity, time-point and location. The treatment of genomic DNA with bisulfite, converting cytosine to uracil, can be used to identify methylated DNA sequences. Microarrays and quantitative real-time PCR assays are frequently used to study the level of gene expression on a genome-wide or focused scale. Beside standard gene expression microarrays, tilling arrays promote the discovery of new transcripts by the annotation of transcriptionally active regions and together with exon arrays, they are suitable for the quantification of alternative splicing events. The combination of ChIP with target detection through arrays (ChIP-on-chip) enables the identification of regulatory elements on a global scale. It can be used to identify *in-vivo *DNA and chromatin binding sites of transcription factors, active promoters as a measure of gene expression and to investigate histone modifications important for chromatin structure. The first application of ChIP-on-chip has been in yeast, and in 2004 the ENCODE consortium has been launched aiming to identify all functional regulatory elements in human [1]. One limitation of ChIP-on-chip is that the discovery of TF binding sites depends on the condition under which a TF of interest is expressed, nuclear and active. Here, double-stranded DNA arrays (protein binding microarrays) provide the method of choice for *in-vitro *binding motif and site discovery in a high-throughput manner. Furthermore, it allows the analysis of TF affinity features in the perspective of nucleotide variations. Protein binding microarrays are less dependent on the availability of specific antibodies as they are mainly performed with tagged TFs. The latter is also feasible for ChIP-on-chip, but here the effect of over-expression needs to be considered.

## Competing interests

The author declares that they have no competing interests.

## Supplementary Material

Additional file 1 **Recommended reading**. Given is an advanced reading list for aspects covered. Click here for file

## References

[B1] The ENCODE Project: ENCyclopedia Of DNA Elements. http://www.genome.gov/ENCODE/.

