# Supplementary material for: Transcriptional regulation at a glance
Source: BMC Bioinformatics. 2007 Sep 27;8(Suppl 6):S2. doi: 10.1186/1471-2105-8-S6-S2 (PMC1995546; doi:10.1186/1471-2105-8-S6-S2)
Supplement: Additional file 1 — Recommended reading. Given is an advanced reading list for aspects covered. [file 1471-2105-8-S6-S2-S1.pdf]

Recommended reading

1. Alberts B: **Molecular biology of the cell**, 4 edn: GS Garland Science Textbooks; 2002.
2. Bantignies F, Cavalli G: **Cellular memory and dynamic regulation of polycomb group proteins**. *Curr Opin Cell Biol* 2006, **18**(3):275-283.
3. Barrera LO, Ren B: **The transcriptional regulatory code of eukaryotic cells--insights from genome-wide analysis of chromatin organization and transcription factor binding**. *Curr Opin Cell Biol* 2006, **18**(3):291-298.
4. Bentley DL: **Rules of engagement: co-transcriptional recruitment of pre-mRNA processing factors**. *Curr Opin Cell Biol* 2005, **17**(3):251-256.
5. Buratowski S: **Connections between mRNA 3' end processing and transcription termination**. *Curr Opin Cell Biol* 2005, **17**(3):257-261.
6. Cara M, Smale ST: **Transcriptional Regulation in Eukaryotes**. New York: Cold Spring Harbour Laboratory Press; 2000.
7. Carthew RW: **Gene regulation by microRNAs**. *Curr Opin Genet Dev* 2006, **16**(2):203-208.
8. Cole CN, Scarcelli JJ: **Transport of messenger RNA from the nucleus to the cytoplasm**. *Curr Opin Cell Biol* 2006, **18**(3):299-306.
9. Conti E, Izaurralde E: **Nonsense-mediated mRNA decay: molecular insights and mechanistic variations across species**. *Curr Opin Cell Biol* 2005, **17**(3):316-325.
10. Cremer T, Cremer M, Dietzel S, Muller S, Solovei I, Fakan S: **Chromosome territories--a functional nuclear landscape**. *Curr Opin Cell Biol* 2006, **18**(3):307-316.
11. Eissenberg JC, Shilatifard A: **Leaving a mark: the many footprints of the elongating RNA polymerase II**. *Curr Opin Genet Dev* 2006, **16**(2):184-190.
12. Elgin SCR, Workman JL: **Chromatin Structure and Gene Expression**, vol. 2: Oxford University Press; 2000.
13. Felsenfeld G, Groudine M: **Controlling the double helix**. *Nature* 2003, **421**(6921):448-453.
14. Fillman C, Lykke-Andersen J: **RNA decapping inside and outside of processing bodies**. *Curr Opin Cell Biol* 2005, **17**(3):326-331.
15. Fischle W, Wang Y, Allis CD: **Binary switches and modification cassettes in histone biology and beyond**. *Nature* 2003, **425**(6957):475-479.
16. Freitag M, Selker EU: **Controlling DNA methylation: many roads to one modification**. *Curr Opin Genet Dev* 2005, **15**(2):191-199.
17. Greive SJ, von Hippel PH: **Thinking quantitatively about transcriptional regulation**. *Nat Rev Mol Cell Biol* 2005, **6**(3):221-232.
18. Harbison CT, Gordon DB, Lee TI, Rinaldi NJ, Macisaac KD, Danford TW, Hannett NM, Tagne JB, Reynolds DB, Yoo J *et al*: **Transcriptional regulatory code of a eukaryotic genome**. *Nature* 2004, **431**(7004):99-104.
19. Huebert DJ, Bernstein BE: **Genomic views of chromatin**. *Curr Opin Genet Dev* 2005, **15**(5):476-481.
20. Kornberg RD, Lorch Y: **Twenty-five years of the nucleosome, fundamental particle of the eukaryote chromosome**. *Cell* 1999, **98**(3):285-294.
21. Kornblihtt AR: **Promoter usage and alternative splicing**. *Curr Opin Cell Biol* 2005, **17**(3):262-268.
22. Latchman DS: **Eukaryotic Transcription Factors**, vol. 4: Elsevier Academic Press; 2004.
23. Lin W, Dent SY: **Functions of histone-modifying enzymes in development**. *Curr Opin Genet Dev* 2006, **16**(2):137-142.

24. Lorch Y, Zhang M, Kornberg RD: **Histone octamer transfer by a chromatin-remodeling complex**. *Cell* 1999, **96**(3):389-392.
25. Margueron R, Trojer P, Reinberg D: **The key to development: interpreting the histone code?** *Curr Opin Genet Dev* 2005, **15**(2):163-176.
26. Nightingale KP, O'Neill LP, Turner BM: **Histone modifications: signalling receptors and potential elements of a heritable epigenetic code**. *Curr Opin Genet Dev* 2006, **16**(2):125-136.
27. Odom DT, Zizlsperger N, Gordon DB, Bell GW, Rinaldi NJ, Murray HL, Volkert TL, Schreiber J, Rolfe PA, Gifford DK *et al*: **Control of pancreas and liver gene expression by HNF transcription factors**. *Science* 2004, **303**(5662):1378-1381.
28. Pasquinelli AE, Hunter S, Bracht J: **MicroRNAs: a developing story**. *Curr Opin Genet Dev* 2005, **15**(2):200-205.
29. Raisner RM, Madhani HD: **Patterning chromatin: form and function for H2A.Z variant nucleosomes**. *Curr Opin Genet Dev* 2006, **16**(2):119-124.
30. Seligson DB, Horvath S, Shi T, Yu H, Tze S, Grunstein M, Kurdistani SK: **Global histone modification patterns predict risk of prostate cancer recurrence**. *Nature* 2005, **435**(7046):1262-1266.
31. Travers A: **An engine for nucleosome remodeling**. *Cell* 1999, **96**(3):311-314.
32. Tsonis PA: **Anatomy of Gene Regulation: A Three-Dimensional Structural Analysis**, vol. 1: Cambridge University Press; 2003.
33. Zlatanova J: **MeCP2: the chromatin connection and beyond**. *Biochem Cell Biol* 2005, **83**(3):251-262.
